# Academics’ perceptions of what it means to be both a parent and an academic: perspectives from an English university

**DOI:** 10.1007/s10734-021-00697-5

**Published:** 2021-03-23

**Authors:** Kayleigh Rosewell

**Affiliations:** Department of Educational Research, Lancaster University, Lancaster, UK

**Keywords:** Higher education, Work and family, Academic roles, Parent roles

## Abstract

This article explores male and female academics’ perceptions of what it means to be both a parent and an academic and the relations between them. Based on an Interpretative Phenomenological Analysis of interviews with 35 academics from a university in England, findings suggest that the way in which academics experience being both a parent and an academic depends upon how they understand the meaning of each of these two roles and what they are trying to achieve within them. These meanings and experiences also appear to differ by gender. Ways in which higher education can offer more targeted and specific support to academic mothers in particular are discussed.

## Introduction

Researchers worldwide have documented gender inequality in higher education (HE) for many decades (e.g., Bhopal, 2020; Coate & Howson, 2014; Morley, 2006). In the United Kingdom (UK), women make up 42% of full-time academic staff (HESA, 2018/19), but they still face barriers to promotion to senior roles (Morley, 2013). This complex problem cannot be attributed to a single cause; however, research shows that motherhood plays a role in gender inequality in HE (Baker, 2012; Dickson, 2019; Wilton & Ross, 2017).

Clearly, it is important to understand the personal and institutional barriers, challenges and successes that academic mothers (and parents) face when attempting to balance work and family, as these will assist in the development of relevant support and policies, which in turn are likely to affect who chooses an academic career, where they choose to work and how they maintain their productivity, satisfaction and well-being (Ward & Wolf-Wendel, 2012). Work-family balance however is only part of the picture. For instance, Dzuriyatun (2020) shows that in the context of New Zealand, the Government’s introduction of work-family policies has failed to provide gender equality. It is, therefore, important to widen our understanding beyond work-family balance. There is no research that has explored what it actually *means* to be both an academic and a parent and the relations between them. The meaning that academics assign to their roles is likely to affect their sense of purpose, motivation and commitment (Day et al., 2006) and influence the ways in which they manage their lives (Henkel, 2000). Without a fuller understanding of the relationship between academic work and parenthood, initiatives that intend to promote equality, diversity and inclusion may be limited and they may well misunderstand, or even reproduce, the problem. In the UK for example, focused interventions that are used to recognise institutions that successfully embed gender equality practices and policies, such as Athena SWAN, can become a tick-box exercise (O’Connor, 2019). Barnard (2017) concluded that even UK departments that are awarded the Athena SWAN gold standard (highest award) often do not tackle the gender inequality that exists in senior positions.

The purpose of this article is to explore the meaning of being an academic and a parent and the relations between them in the context of England. The focus on men as well as women is not to minimise the challenges that academic mothers face, or to suggest that men and women have the same experiences of work and family; rather, it allows us to highlight the different ways in which academic and parent roles are understood and experienced. This can enhance our understanding of work and family, and provide an additional perspective on men and women’s academic careers.

## What the literature tells us about academic work and parenthood

There is a growing body of international research on academic work and motherhood, particularly in the United States (US) (Wolf-Wendel & Ward, 2006; Gilbert, 2008; Philipsen, 2008; Sotirin, 2008; Stinchfield & Trepal, 2010; Connelly & Ghodsee, 2011; O’Brien Hallstein & O’Reilly, 2012; Trepal & Stinchfield, 2012; Castaneda & Isgro, 2013; Ward & Wolf-Wendel, 2004, 2012, 2016), and also in Europe (Lutter & Schröder, 2020; Thun, 2019) and England in particular (Amsler & Motta, 2019; Jenkins, 2020; Leonard & Malina, 1994; Munn-Giddings, 1998; Raddon, 2002). This literature focuses on issues relating to work-family balance and on the ways in which becoming and being a mother affect academic work and careers in some positive, though mainly in negative, ways, with many of the findings being similar across different studies.

Academic work is demanding and unending; and measures of success are linked to obtaining research funding, publishing research and attending conferences, all of which are considered to be crucial career-building activities that are often done during evenings and weekends (Raddon, 2002). This poses particular challenges for mothers, who are often responsible for a greater share of childcare and home care (Misra et al. 2012; O’Meara & Campbell, 2011; Thun, 2019; Wolf-Wendel & Ward, 2006). Further, the notion of the successful academic (Raddon, 2002) is in direct conflict with discourses of good mothering (Raddon, 2002; Johnston and Swanson, 2006).

Although there is less research, studies on academic fathers suggest that they too experience similar challenges when combining work and family life (Philipsen & Bostic, 2010; Marotte et al., 2011; Dillon, 2012) and that they face pressure when attempting to be both a successful academic and a good father (Duan et al. 2010; Gould & Lovato, 2019; Reddick et al. 2012; Sallee, 2012). However, the literature is clear that the challenges are greater for mothers, due to the physical demands of pregnancy and childbirth, gendered expectations of family obligations, and the ongoing inequality with which working women take on the ‘second shift’ (Hochschild, 1989) of home care and childcare (Misra et al. 2012; O’Meara & Campbell, 2011; Thun, 2019; Wolf-Wendel & Ward, 2006), even within dual-career households (Erickson, 2005). According to Ruddick (1989), mothers also do more of the emotional labour and ‘maternal thinking’, which is guided by an interest in their child’s preservation, growth and acceptability. Furthermore, socially constructed and gendered notions of women as caregivers may also contribute to differences in the academic work carried out, as females tend to have higher teaching, administrative and service loads, which may involve considerable emotional labour, yet this work is often undervalued and simply reinforces gender inequality in HE (Flaherty, 2020; Leathwood & Hey, 2009; Rickett & Morris, 2021).

Although the current research was carried out prior to the COVID-19 pandemic, recent studies show that the pandemic has exacerbated these inequalities further in both academia and the home (Flaherty, 2020; Willey, 2020). Although men are not immune to the impact of the pandemic, Flaherty (2020) suggests that women are now having to devote their time to home schooling their children and doing the extra household work that has been created by families spending more time in the home, and that they are also taking on a greater share of teaching and producing fewer single-authored journal submissions, whilst submissions from men have increased. Academic mothers, therefore, suffer what has been called the ‘motherhood penalty’ (Baker, 2012).

On a more positive note, studies on both mothers (Gilbert, 2008; Trepal & Stinchfield, 2012; Ward & Wolf-Wendel, 2004; Willey, 2020; Wolf-Wendel & Ward, 2006) and fathers (Reddick et al. 2012) suggest that becoming a parent can help to put the stress of academic work into a healthier perspective and that they juggle their roles by becoming more efficient. Academic parents may adopt different strategies as a way to juggle their roles. Furedi (2003) shows that academic fathers successfully balance their roles by compartmentalising their lives. Other research on academic fathers (Dillon, 2012) and on academic mothers (Goodier, 2012; Green, 2003; Minaker, 2012; Pillay, 2012; Raddon, 2002; Willey, 2020) found that academics who are also parents experience a greater sense of balance when their roles feel connected. However, this does not necessarily mean that academics switch between different roles that have different meanings, or that they behave differently according to which role they are performing. As Halpern (2008, p. 58) notes, ‘real people live one integrated life, not two’; people do not leave one sphere and enter another.

What the literature fails to consider is what being an academic and a parent *actually mean* and the relations between these two roles. Existing literature tends to present the *experience* of being an academic and a mother and a father in similar ways, despite showing that the effects of motherhood and fatherhood differ. This is problematic because it implies (1) that being an academic means the same to all academics; (2) that being a parent means the same to all academics; and finally, (3) that the relationship between the two roles is qualitatively the same for all academics, even if quantitatively the time spent on them is different or the impact of parenthood is different for men and women.

## Methods and methodology

This article draws on Interpretative Phenomenological Analysis (IPA) as a qualitative methodology that focuses on personal lived experience (Smith et al., 2009). One of the strengths of IPA is that it is underpinned by phenomenology, hermeneutics and idiography. The phenomenological and hermeneutic lens of IPA is highlighted in its focus on an interpretative process, which involves the researcher trying to make sense of the participant who is trying to make sense of their own lived experiences, as well as on the ‘hermeneutic circle’ (Smith, 2007, p. 5) of moving back and forth between the part and the whole so that the data can be thought about in different ways (Smith et al., 2009). The idiographic focus of IPA lies in its commitment to giving a detailed account of each participant in turn before making more general claims.

In accordance with IPA, semi-structured interviews were used, as this allows participants to offer rich and detailed accounts of their lived experience. The interview schedule focused on what a typical week as a parent and an academic involves, likes, dislikes, values and goals in relation to each role, and perceptions about what it actually means to be both a parent and an academic. Interviews typically lasted an hour and were recorded digitally and then transcribed.

It is important to recognise that whilst a ‘particular position of an institutional site(s) can be read across national and global hierarchies’ (Clegg, 2008, p. 332), the contextual and structural differences between HE institutions play a significant role in shaping academics’ experiences of their work (Clarke, 1987; Trowler, 1998) as well as their experiences of work-family balance (Wolf-Wendel & Ward, 2006). Participants were recruited from a single university in England. Universities in England are part of a single system and are officially independent organisations. The institution where interviewees work is a pre-1992 campus university, but outside of the Russell Group, and is a teaching and research-focused institution, although it is particularly research-led. It is regularly ranked highly in each of the UK’s major university league tables and performs well in the Research Excellence Framework (REF) and in student satisfaction surveys. The university offers a package of work-family policies, including paid maternity and paternity leave which can be transferred or shared under a shared parental leave policy, additional leave if needed and flexible working arrangements. There is also a childcare facility on campus. This is broadly representative of work-family policies at universities in England.

Participants were recruited using a purposive sampling method. The criteria for inclusion in this research were that participants had to be employed as ‘academic staff’ (lecturer to professor) and have children. Participants were invited to take part in this research by an initial email, which contained some brief information about the study. The study was also advertised through the childcare facility on campus.

Thirty-five interviews were conducted with male and female academics of different ages, disciplines, academic roles and career stages (see Table 1). All have been assigned a pseudonym to protect their identity. Thirty-two participants were aged between 30 and 49. Most participants were in heterosexual marriages, with only one being in a non-heterosexual relationship and three being single mothers. Twenty-six participants had children aged 11 or under. The number of those categorised as an early (5 years or less experience), middle (6–10 years’ experience) or late (11 or more years’ experience) career academic was relatively even, although the smallest category was mid-career academics. Only four participants were readers (three males and one female) and only two were professors (both male). Only three participants worked part-time and all three were females. The participants’ disciplinary backgrounds have been characterised using Biglan’s (1973a, b) typology of academic disciplines to protect their identity. However, this article does not explore disciplinary or career stage differences or the differences according to full- or part-time working hours, relationship status, number, age or gender of children. Future analyses will consider these differences in greater detail.

**Table 1:** Demographic variables

Discipline	Pseudonym	Age	Position	Career stage
Hard applied	John	40–49	Senior lecturer	Late
	Andrew	30–39	Professor	Late
	Damon	40–49	Senior lecturer	Late
	Jack	40–49	Lecturer	Late
	Nathan	30–39	Lecturer	Early
	Sophie	40–49	Senior lecturer	Middle
	Miranda	40–49	Senior lecturer	Late
	Samantha	40–49	Lecturer	Early
	Jennifer	40–49	Senior lecturer	Late
Hard pure	Lewis	50–60	Senior lecturer	Late
	Jake	40–49	Senior lecturer	Middle
	Leon	40–49	Reader	Middle
	Rick	40–49	Senior lecturer	Middle
	Vicky	40–49	Senior lecturer	Late
	Kate	40–49	Lecturer	Early
	Zoe	30–39	Lecturer	Early
	Louise	30–39	Lecturer	Early
Soft applied	Robert	30–39	Lecturer	Early
	Mark	30–39	Lecturer	Middle
	Max	40–49	Lecturer	Early
	Graham	30–39	Lecturer	Early
	Michelle	30–39	Lecturer	Late
	Hayley	30–39	Lecturer	Early
	Kirsty	30–39	Lecturer	Early
	Jade	30–39	Senior lecturer	Late
Soft pure	Michael	40–49	Reader	Late
	Richard	50–60	Senior lecturer	Late
	Dylan	40–49	Professor	Late
	Mathew	50–60	Reader	Late
	Jason	30–39	Lecturer	Middle
	Christopher	30–39	Lecturer	Middle
	Rebecca	30–39	Lecturer	Early
	Sandra	40–49	Reader	Middle
	Natalie	40–49	Senior lecturer	Middle
	Deborah	40–49	Lecturer	Late

The data was analysed using Smith et al’s. (2009, p. 79) six steps to IPA, which involves ‘moving from the particular to the shared, and from the descriptive to the interpretative’. The first step involved reading and re-reading the transcript, which allowed me to enter a phase of ‘active engagement’ with the data (Smith et al., 2009, p. 82). The second step focused on developing descriptive comments that remained close to the participants’ own words. This is where specific ways in which participants understand being an academic and a parent emerged. To support this process, I developed pen portraits for each interviewee (usually two to three pages) which focused on developing an overall picture of what it means to be both an academic and a parent, and what *matters* most to each participant. Pen portraits can allow interviewees to become ‘more alive and present in our write up’ (King & Horrocks, 2010, p. 139). The pen portraits provided an illustration of each participant’s overriding central story, which highlighted the consistency in the articulation of the meaning of being an academic and a parent within individual interviews. The third step involved a process of turning notes into themes. The fourth step focused on identifying patterns between emergent themes and developing ‘super-ordinate’ themes (Smith et al., 2009, p. 96). The fifth step involved moving onto the next transcript and repeating the process as described above. Once a set of super-ordinate themes had been developed for each participant, the final step centred on searching for patterns and developing a final set of themes that firmly remained close to participants’ own words.

As multiple interpretations can be made of the same data, I focused on validity rather than reliability and drew on Kvale and Brinkmann’s (2009) notions of communicative and pragmatic validity and quality of craftsmanship. The focus was on an interpretation of the data that is defensible and the extent to which the research outcomes reflected the phenomenon being studied (Åkerlind, 2005; Kvale & Brinkmann, 2009). Communicative validity develops when the research methods and final interpretation of the outcomes are accessed by the relevant research community through seminars, conference presentations and peer-reviewed journals and are seen as appropriate (Kvale & Brinkmann, 2009). Valid knowledge is established when the audience can answer new questions and advance conversations in the research area (Kvale & Brinkmann, 2009). Finally, pragmatic validity refers to the extent to which the research findings are viewed as meaningful, useful and relevant to their intended audience (Kvale & Brinkmann, 2009).

To explore the relations between the meaning of being an academic and a parent, this article draws on analysis from research reported in a previous article on what it means to be an academic (see Rosewell & Ashwin, 2018). The three different meanings of being an academic were:

1. Being a teacher
2. Being a researcher as a creative process, as a process of discovery or as professional recognition
3. A general view of being an academic as either self-focused or as providing a contribution.

## Findings

Participants identified four central meanings of being a parent: (1) as ensuring that children become a particular type of person; (2) as allowing children to become who they are; (3) the benefits of being a parent; and (4) as being part of their individual sense of self. Within each of these meanings, all participants defined parenthood as a sense of responsibility to raise children from childhood to adulthood, to care for and nurture them, and to ensure that they are healthy, safe, happy and educated.

### Ensuring children become a particular type of person

Seven participants viewed the meaning of being a parent as to ensure children become a particular type of person, by educating children in a *correct* and *proper* way so that they grow up to be good people with the *right kind* of manners and attitude:

I think I’m becoming more aware that, for me, being a parent is making sure that my children can go into a certain type of job. Not necessarily an academic one, but that kind of job. It’s difficult to say what that means because I’m not trying to say that one type of job is better than another, but I’m trying to make sure my children go into a job. I can’t really explain. (Mark).

This shows the vision which Mark held of who his children would become and what they would aspire to be in the future, and his attempt to shape and steer them in and towards a particular direction. Although the other six participants did not discuss ideas around particular jobs, they all held a particular vision of their children and saw their role as to shape and steer them in these ways. These participants expressed a difficulty in articulating these meanings, as is demonstrated by Mark’s views in particular. This difficulty appeared to stem from wanting to avoid passing judgement on different approaches to parenting (and professions in Mark’s case), and seems to represent a perceived moral code regarding appropriate parenting.

These participants also appeared to draw on ideas around asserting a hierarchical position. For instance, Rebecca defined her role as ‘being a figure of authority, the one who is in charge’ and whilst she felt that her association with parenting was ‘not entirely positive’, this stemmed from the young age of her children and her perception that they were ‘kind of at that naughty stage’. Similarly, Max explained: ‘it’s my job to teach my boys to become men’, perhaps because his children ‘are getting older’. These views were often connected to the age and gender of the children.

Although these participants saw being a parent as to ensure children become a particular type of person, this is not to say that they were not concerned with their children’s well-being and happiness, but this was seen as something that all parents want for their children rather than as their central parental role.

### Allowing children to become who they are

Nine interviewees defined being a parent as allowing children to become who they are by supporting them to achieve their full potential, goals and dreams and by equipping them with the skills to live happy and successful lives in the ways that they choose. These participants expressed the importance of allowing children to create their own directions and their own ideas about their sense of self.

These participants attached less significance to asserting a hierarchical position and more to creating a friendship that relied on mutual understanding and respect, a view that stemmed from participants’ experiences of their own childhood:

I remember my parents locking my brother in his room telling him “you must do your homework” and acting as that figure of authority. I think the more they tried to control him, the more he rebelled. So, it’s more important for me to be a friend to mine and allow them to make their own decisions and learn from their mistakes. I do try to give them that responsibility to learn from their own mistakes. (Sophie).

Although these participants felt it was important to help children to become *good* people, this was perceived as something that all parents want rather than central to their parental role.

### The benefits of being a parent

Thirteen participants focused on the benefits of becoming a parent, in terms of either the sense of purpose, fulfilment, reward and happiness that they felt (eight participants) or the personal growth and development that they had experienced since becoming a parent (five participants).

These accounts tended to be concise and involved little description beyond their immediate personal gain from being a parent. However, those who focused on the sense of fulfilment from being a parent discussed their childhood and growing up in large families and the happiness this has brought to their lives, which could account for a sense that their lives were complete now that they had built their own family. Only Deborah gave concrete examples of the skills that she had learnt from becoming a parent, which included a new sense of ‘understanding, tolerance and empathy towards others’. Other participants felt that they had gained a new outlook or perspective on the world, but expressed difficulty in articulating this change.

### Being a parent as part of the individual sense of self

Five participants defined being a parent as part of their individual sense of self. Although this view was also present in other accounts, it was not seen as the core of being a parent in the same way. These participants experienced difficulty in articulating the meaning of being a parent beyond the view that it was part of their sense of self. Hayley explained: ‘I don’t really think about being a mother; I think about my son; I’ve never really thought about what it means to be a mother, it’s *just* instinctive’. Similarly, Damon said: ‘I think I’m clear about my work role, but being a parent is just part of me; it’s less clear in that respect’. These comments give a sense of a child-centred notion of being a parent and a view of parenting as natural and instinctive, which seems to contribute to the difficulty of articulating the meaning.

Interestingly, the parents in this group were the only interviewees to reflect on societal definitions of being a parent:

This might be a gender-specific thing, but for me it’s also important to communicate that I’m not just a parent; yes, I like being a parent and I love my son to bits, but I’m not just a parent. I think friends, family, especially male family members, assumed that I would change once I became a parent and I would just care about the baby, but *I* still matter. The last time I checked, my PhD wasn’t in nappy changing, and my academic identity means the same thing to me today as it did prior to having a child. So, it’s important that people don’t just see me as a parent, because I don’t like this expectation in society that mothers are only mothers and live through their baby. (Jade).

I think there’s a lot of crap talked about parenthood, what’s good parenting, bad parenting, societal definitions about what parents should and shouldn’t be doing. Increasingly we do try to rebel against such definitions. (Damon).

The above comments suggest that, although participants experienced difficulty in defining the meaning of their individual parental role, they appeared to be able to discuss societal meanings and representations of parenthood with greater ease and to deconstruct those meanings.

### The relations between what it means to be a parent and gender

Academics’ perceptions of the meaning of being a parent appeared to relate to gender. As shown in Table 2, the men tended to define being a parent in terms of the benefits, particularly the sense of purpose, fulfilment, reward and happiness that they experienced since becoming a parent, and only one male viewed being a parent as part of their individual sense of self. The women tended to focus on being a parent as allowing children to become who they are and as part of their individual sense of self. Only two of the women viewed being a parent as to ensure children become a particular type of person, and three of the four women that focused on the benefits of being a parent did so in terms of the personal growth and development that they had experienced themselves since becoming a parent.

**Table 2:** The relations between what it means to be a parent and gender

Being a parent	Men	Women
Ensuring children become a particular type of person	5	2
Allowing children to become who they are	4	5
The benefits of being a parent (sense of purpose, fulfilment, reward and happiness)	7	1
The benefits of being a parent (personal growth and development)	2	3
Being a parent as part of the individual sense of self	1	5

### The relations between what it means to be an academic and what it means to be a parent

When looking closely at the accounts of being an academic (Rosewell & Ashwin, 2018) and a parent, there appeared to be a relationship in some cases (see Table 3). Three of the four participants that gave an account of being a researcher as a creative process and six of the twelve that gave an account of a general view of being an academic as self-focused also gave an account of their parental role in relation to the benefits. Five of the six academics that defined being a researcher as professional recognition also defined being a parent as ensuring children become a particular type of person. All participants that constructed a general view of being an academic as providing a contribution also defined being a parent as allowing children to become who they are.

**Table 3:** The relations between what it means to be an academic and what it means to be a parent

What it means to be a parent	What it means to be an academic					
	Teacher	Researcher as a creative process	Researcher as a process of discovery	Researcher as professional recognition	Academic as self-focused	Academic as providing a contribution
To ensure children become a particular type of person			1	5	1	
To allow children to become who they are	1		1		1	6
Benefits of being a parent	3	3		1	6	
Part of the individual sense of self		1	1		4	

## Discussion

The purpose of this article was to explore academics’ perceptions of what it means to be an academic and what it means to be a parent and the relations between them. Previous research gives no attention to these meanings, focusing primarily on work-family balance and the impact that becoming a parent has on academic careers, particularly on women’s careers. The current research adds to the international debates on academic work and parenthood by examining them in the context of England and demonstrating not only that work-family balance is only part of the picture (Dzuriyatun, 2020) but also that the meaning of being an academic and a parent plays an important role in shaping men and women’s academic career trajectories.

So why is this important? On the one hand, the literature is clear that academic mothers are more negatively affected by parenthood than academic fathers. This is due not only to the unequal division of labour in the home but also to the way that roles are allocated in academia. On the other hand, the actual challenges and successes that men and women experience when balancing work and family are presented as somewhat similar. The current findings show that the *experience* of being an academic and a parent are in fact different for men and women because the *meaning* of these roles differs for men and women. This diversity of lived experience is overlooked in the existing literature, which has limited our understanding of the impact that becoming and being a parent has on women and men’s academic careers. In accordance with other research, it is the meaning of academics’ roles that influences their sense of purpose (Day et al., 2006) and the ways in which they manage their lives (Henkel, 2000). It is not just about work-family balance; it is also about the different meanings, intentions, goals and aspirations of, and in, their roles.

The different ways in which men and women define the meaning of being an academic have been detailed elsewhere (see Rosewell & Ashwin, 2018) and the current findings suggest that the meaning of being a parent also appears to be gendered. Being a mother tends to be all-encompassing in the way that it is more commonly seen as being part of the individual self, and the meaning is often more child than parent-centred. Being a father tends to be an individualistic and parent-centred experience. This suggests that women’s roles are perhaps more likely to incorporate more of the emotional labour (Ruddick, 1989). However, who these academics want their children to be/become is interesting. As already discussed, women tend to see being a parent as part of the individual self or as allowing children to become who they are. Whilst men tend to see being a parent in terms of personal benefit, the number of men that do define being a parent as ensuring children become a particular type of person or as allowing children to become who they are, is relatively even. Ruddick (1989) argues that one aspect of ‘maternal thinking’ is guided by a mother’s desire to shape children into the type of person that is socially accepted. The current findings, however, show that fathers are more likely than mothers to define being a parent in this way and that both men and women draw on notions of parenthood that require maternal thinking, but the nature of this thinking differs for men and women, with a tendency for women to be concerned with children becoming who they are and men with social acceptability.

The relations between the meaning of being an academic (Rosewell & Ashwin, 2018) and the meaning of being a parent highlight a significant and telling story. The current outcomes show that, for some academics, the overarching meaning of being a parent and being an academic and what they are trying to achieve in these roles are similar, and that there is consistency in the ways that they approach these different aspects of their lives (Halpern, 2008). In accordance with previous literature, academic and parent roles are connected (Dillon, 2012; Goodier, 2012; Green, 2003; Minaker, 2012; Pillay, 2012; Raddon, 2002; Willey, 2020), and in ways that dismiss the separation and competition that the concept of work-family balance implies.

**Fig. 1:**
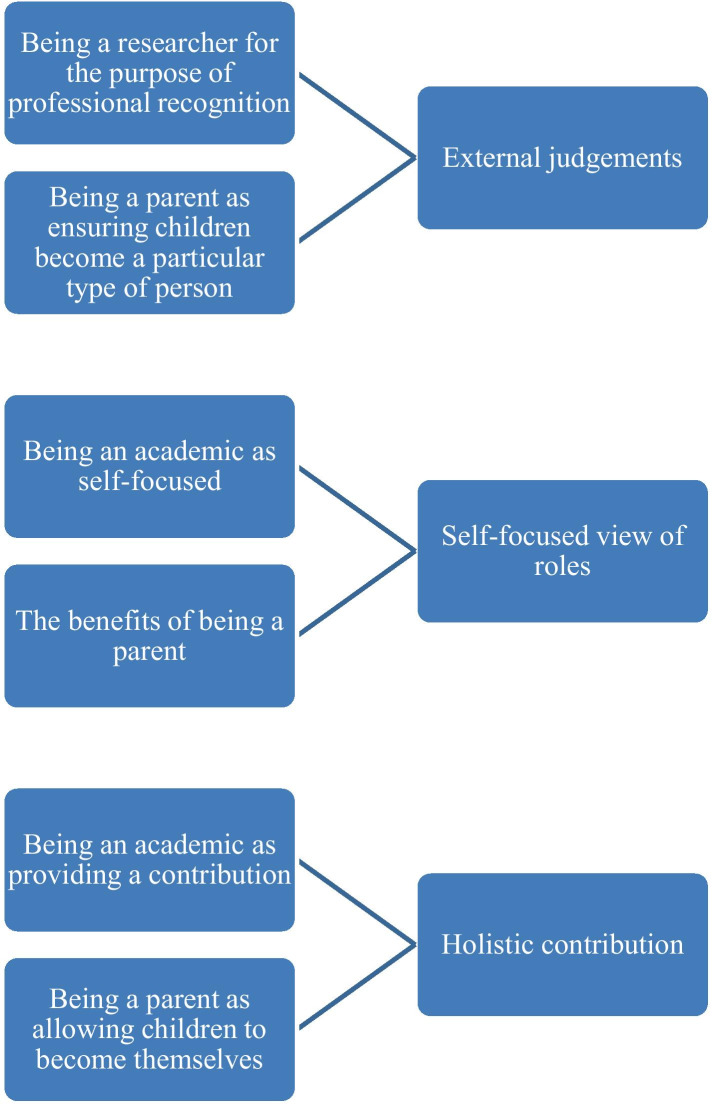
The relations between what it means to be an academic and what it means to be a parent

Figure 1 highlights three overarching relations between being an academic and a parent: external recognition, self-focused roles and holistic contributions. The academics that define being a parent as ensuring children become a particular type of person are likely to define being an academic as a researcher for the purpose of professional recognition. This suggests that the academics who are concerned with external recognition for their work and career may also want their children to be recognised as a particular type of person. This gives a sense of primary concern with external judgements. The academics that define being a parent in terms of the benefits are likely to define being an academic as being a researcher as a creative process and as being an academic in general as self-focused. This suggests that those that focus on what they get out of being an academic also tend to focus on what they get out of being a parent. The academics that define being a parent as allowing children to become who they are are likely to define being an academic as providing a contribution. This suggests that the academics who are focused on providing a contribution in their career to wider society and/or to the lives of their students also want their children to find themselves.

Importantly, the relations between the meaning of being an academic and a parent appear to relate to gender. Those with an overarching view of their academic and parent roles as self-focused or as centred on external recognition tend to be male and those with an overarching aim to provide holistic contributions in their roles and a view of their roles as part of the individual self tend to be female. These outcomes are significant and contribute to the research that highlights the role that motherhood plays in gender inequality in HE (Baker, 2012; Dickson, 2019; Wilton & Ross, 2017). An understanding of being an academic and a parent as self-focused or as concerned with external recognition centres on particular goals that tend to be individualistic and career and parent-centred. This differs quite significantly to an understanding of being an academic and a parent that centres on providing a contribution that is driven towards others in both academic work (students, colleagues, society) and motherhood (children). Consequently, male academics are perhaps more able to focus on, and experience, research as professional recognition, whilst for female academics, research is just another component of their work. Further, research as an activity intended to provide a contribution is likely to require a different type of energy, thinking and labour than research as an activity primarily to obtain recognition and career advancement. Furthermore, if female academics are more likely to see being a parent and academic as part of their individual self, this may result in them being less able to separate or compartmentalise their lives in ways that men are perhaps able to do (Furedi, 2003). It would appear that men define their academic roles in ways that align more significantly with notions of the successful academic (Raddon, 2002; Wolf-Wendel & Ward, 2006) and this furthers our understanding of men and women’s academic career trajectories.

It is important to recognise that what might appear to be personal choice (Dickson, 2019; Wolf-Wendel & Ward, 2006) about the ways in which men and women articulate the meaning of being an academic and a parent and their aspirations within each role may actually be influenced by normative structures in HE and wider society. If the intentions and goals of academic mothers and fathers are indeed personal choices, universities should consider the role that research and publications play in promotions criteria and the work demands that this encourages. Universities should also be striving to tackle the well-documented gender inequalities in academic work and the ‘motherhood penalty’ (Baker, 2012), as these clearly contribute to the different career trajectories of male and female academics.

## Conclusion

This article highlights the different ways in which men and women define their academic and parent roles and what they are trying to achieve in them. Recognising that women struggle to balance academic work and motherhood due to an unequal distribution of labour in both academia and the home does not go far enough in tackling some of the challenges that women face. This has important implications for how academics, and in particular female academics, are supported. Whilst work-family balance support and policies are essential, achieving success, satisfaction and happiness as an academic is not just about gaining work-family balance; it is also about being recognised and valued, and part of this stems from understanding the role that identities, aspirations, goals and intentions play in career and life trajectories.

Although the outcomes in this article are based on a small-scale study, it provides a useful contribution to the international debates in the research on academic work and parenthood and can be used to (re)consider the different ways to engage and support the diverse lived experiences of academics that exist within and outside of institutions. Further, whilst the findings are based on the perceptions of academics working in a single university in England, they do allow us to draw some conclusions about the English HE system and other contexts where similarities exist. The findings are relevant to HE staff that are concerned with the recruitment, retention, job satisfaction and well-being of academics, as well as to anyone tackling issues of gender inequality and promoting equality, inclusion and diversity, which are important globally.

This article advocates for a move away from gender-neutral discourses, policies and support that may ignore and even normalise gender inequalities in academic work and beyond, including normative structures that define success in academia and how work-family balance is supported. It is vital that universities not only tackle the unequal distribution of academic work that may constrain women’s academic choices but also truly recognise and value women’s academic roles and their goals and intentions, as these may differ to those that are currently valued in HE. Academic mothers must be considered in policies in academia as they have important roles to play in disrupting such normative academic structures.

It is important to recognise the limitations of this article. As already discussed, this article is based on a single research-intensive institution in England. In the English HE system, some institutions are more research focused than others. Whilst the pressures associated with reaching tenure are not relevant to universities in England as to those in the US, the relations between research and career advancement in the English HE system are similar to those in institutions in America, whose research tends to dominate in the literature on academic work and family. The participant sample in this study was under-represented by academics at the top of the career ladder, and those that were in these positions tended to be male. Further, the sample included a small number of same-sex and single parents and those that worked part-time, all of which were also female. An area for future research would therefore be to widen the sample in terms of diversity of academics, as well as different types of higher education institutions within England, such as those that are teaching or vocationally focused, as well as to widen the research in terms of national and global comparisons.

Greater understanding of what it means to be an academic and a parent and the relations between them will enable HE policy makers, academic line managers and human resources professionals working in HE to provide much-needed gender-specific changes that primarily will better support mothers, but which will also benefit fathers and those academics with additional care responsibilities.
